# Distribution of ELOVL4 in the Developing and Adult Mouse Brain

**DOI:** 10.3389/fnana.2017.00038

**Published:** 2017-05-01

**Authors:** David M. Sherry, Blake R. Hopiavuori, Megan A. Stiles, Negar S. Rahman, Kathryn G. Ozan, Ferenc Deak, Martin-Paul Agbaga, Robert E. Anderson

**Affiliations:** ^1^Department of Cell Biology, University of Oklahoma Health Sciences CenterOklahoma City, OK, USA; ^2^Oklahoma Center for Neuroscience, University of Oklahoma Health Sciences CenterOklahoma City, OK, USA; ^3^Department of Pharmaceutical Sciences, University of Oklahoma Health Sciences CenterOklahoma City, OK, USA; ^4^Dean McGee Eye Institute, University of Oklahoma Health Sciences CenterOklahoma City, OK, USA; ^5^Department of Ophthalmology, University of Oklahoma Health Sciences CenterOklahoma City, OK, USA; ^6^Harold Hamm Diabetes Center, University of Oklahoma Health Sciences CenterOklahoma City, OK, USA; ^7^Department of Geriatric Medicine, Reynolds Oklahoma Center on Aging, University of Oklahoma Health Sciences CenterOklahoma City, OK, USA

**Keywords:** very long chain fatty acids, spinocerebellar ataxia, stargardt-like macular dystrophy, seizure, hippocampus, cortex, cerebellum

## Abstract

ELOngation of Very Long chain fatty acids (ELOVL)-4 is essential for the synthesis of very long chain-fatty acids (fatty acids with chain lengths ≥ 28 carbons). The functions of ELOVL4 and its very long-chain fatty acid products are poorly understood at present. However, mutations in *ELOVL4* cause neurodevelopmental or neurodegenerative diseases that vary according to the mutation and inheritance pattern. Heterozygous inheritance of different *ELOVL4* mutations causes Stargardt-like Macular Dystrophy or Spinocerebellar Ataxia type 34. Homozygous inheritance of *ELOVL4* mutations causes more severe disease characterized by seizures, intellectual disability, ichthyosis, and premature death. To better understand ELOVL4 and very long chain fatty acid function in the brain, we examined ELOVL4 expression in the mouse brain between embryonic day 18 and postnatal day 60 by immunolabeling using ELOVL4 and other marker antibodies. ELOVL4 was widely expressed in a region- and cell type-specific manner, and was restricted to cell bodies, consistent with its known localization to endoplasmic reticulum. ELOVL4 labeling was most prominent in gray matter, although labeling also was present in some cells located in white matter. ELOVL4 was widely expressed in the developing brain by embryonic day 18 and was especially pronounced in regions underlying the lateral ventricles and other neurogenic regions. The basal ganglia in particular showed intense ELOVL4 labeling at this stage. In the postnatal brain, cerebral cortex, hippocampus, cerebellum, thalamus, hypothalamus, midbrain, pons, and medulla all showed prominent ELOVL4 labeling, although ELOVL4 distribution was not uniform across all cells or subnuclei within these regions. In contrast, the basal ganglia showed little ELOVL4 labeling in the postnatal brain. Double labeling studies showed that ELOVL4 was primarily expressed by neurons, although presumptive oligodendrocytes located in white matter tracts also showed labeling. Little or no ELOVL4 labeling was present in astrocytes or radial glial cells. These findings suggest that ELOVL4 and its very long chain fatty acid products are important in many parts of the brain and that they are particularly associated with neuronal function. Specific roles for ELOVL4 and its products in oligodendrocytes and myelin and in cellular proliferation, especially during development, are possible.

## Introduction

Lipids play many important roles in the central nervous system (CNS) and are critical to CNS health and function. Recently, lipids have been recognized as important regulators of synaptic function (Marza and Lesa, 2006; Marza et al., 2008; Brodde et al., 2012) and disruption of synapse-associated lipid metabolism can compromise synaptic function and lead to disease (Marza and Lesa, 2006). However, our understanding of lipid metabolism and function at the synapse, particularly mammalian synapses, is limited at present, despite their importance in health and disease.

The fatty acid elongase, ELOngation of Very Long chain fatty acids-4 (ELOVL-4), is the only known enzyme for synthesis of very long chain saturated and polyunsaturated fatty acids (VLC-SFA and VLC-PUFA, respectively; chain length ≥ 28 carbons; Agbaga et al., 2008, 2010; Logan et al., 2013, 2014), and is expressed in only a few tissues (brain, retina, skin, testes, Meibomian glands; Poulos et al., 1987; Mandal et al., 2004; McMahon et al., 2014). In the skin, VLC-SFA are components of several sphingolipids that provide the skin water barrier that protects animals from dehydration (Cameron et al., 2007; Li et al., 2007; McMahon et al., 2007; Vasireddy et al., 2007). In the retina, VLC-PUFA are found only in phosphatidylcholine and are enriched in the outer segment membranes of photoreceptors (Aveldano, 1987; Aveldano and Sprecher, 1987; Rotstein et al., 1996). Although VLC-PUFA in phosphatidylcholine have been reported in rat and human brain (Poulos et al., 1988; Robinson et al., 1990), our lipidomics analyses of mouse brain found only VLC-SFA in phosphatidylcholine in the brain (Hopiavuori et al., 2017).

ELOVL4 and its VLC-fatty acid products are critical to proper neuronal and synaptic function in the retina and CNS. To date, nine different mutations in the human ELOVL4 gene have been identified that cause neurological disorders (Bernstein et al., 2001; Edwards et al., 2001; Zhang et al., 2001; Aldahmesh et al., 2011; Cadieux-Dion et al., 2014; Mir et al., 2014; Bourassa et al., 2015; Ozaki et al., 2015; Agbaga, 2016). Homozygous mutations in human *ELOVL4* cause neurological disorders characterized by seizures, intellectual disability, and neurodegenerative disease (Aldahmesh et al., 2011; Mir et al., 2014). Several heterozygous mutations in human *ELOVL4* have been identified that cause autosomal dominant spinocerebellar ataxia (type 34; SCA34) and/or erythrokeratodermia variabilis (EKV) with no significant retinal phenotype (Cadieux-Dion et al., 2014; Bourassa et al., 2015; Ozaki et al., 2015). Other heterozygous *ELOVL4* mutations cause autosomal dominant Stargardt-like macular dystrophy (STGD3) without any skin or CNS phenotype (Bernstein et al., 2001; Edwards et al., 2001; Zhang et al., 2001).

*Elovl4* is developmentally regulated at the genomic level in the brain, with expression beginning at late embryonic stages, peaking around postnatal day 1 (P1), and then declining in expression by P30, after which it appears to be maintained at a steady-state level (Mandal et al., 2004). This pattern of expression and the linkage of *ELOVL4* mutations to human disease suggest that ELOVL4 and its VLC-fatty acid products have important but as yet unknown functions in the developing and mature brain. Importantly, the spatial and temporal patterns of ELOVL4 expression in the developing and mature brain are unknown. To better understand the roles that ELOVL4 and its VLC-fatty acid products may play in the brain, we mapped ELOVL4 distribution in the mature and developing mouse brain by immunohistochemistry in combination with neuron and glia-specific markers.

## Materials and methods

### Animals and tissue preparation

The expression of ELOVL4 was examined in the brains of wildtype C57BL6 mice collected at embryonic day 18 (E18), and at postnatal days (P) 10, P19–21, and P60. Brains from a total of 23 animals were examined (*n* = 5 at E18; *n* = 4 at P10; *n* = 9 at P19–21; *n* = 4 at P60). Animals were maintained in a pathogen-free barrier facility on a 12 h on:12 h off daily light cycle daily (~150 lux), with food and water available *ad libitum*. All animal procedures were approved by the University of Oklahoma Health Sciences Center Institutional Animal Care and Use Committee and conformed to the National Institute of Health Guide for the Care and Use of Laboratory Animals, the Association for Research in Vision and Ophthalmology Resolution on the Use of Animals in Research, and US Public Health Service guidelines.

To collect brains from E18 pups, dams were euthanized by cervical dislocation and then decapitated. Pups were then isolated from the uterus and the top of the skull was carefully removed and the brain was extracted. Postnatal animals were euthanized by cervical dislocation followed by decapitation, and the brains were removed from the skull. Brains were placed on an ice-cold aluminum block and hemisected. One hemisphere from each animal was embedded unfixed in Optimal Cutting Temperature medium (OCT; Sakura Tissue Tek; VWR, West Chester, PA) and frozen by placing the tissue on an aluminum plate half submerged in liquid nitrogen to freeze the OCT. The other hemisphere was collected for biochemical analyses. Embedded brains were stored at −80°C. Frozen sections (10–15 μm thickness) were prepared on a cryostat and collected onto Superfrost Plus slides (Fisher Scientific, Pittsburgh, PA) and stored at −20 to −30°C until used.

### Immunolabeling and imaging

To perform immunolabeling, cryosections of unfixed brains cut in the sagittal plane were thawed and immersed in 100% methanol at −30°C for 20 min to improve ELOVL4 labeling (Agbaga et al., 2008), rinsed in distilled water, and then rinsed in Hank's Buffered Salt Solution (HBSS). In some experiments cryosections were subjected to antigen retrieval in 10 mM citrate buffer (pH 6.0; heated to 95°C) for 30–60 min prior to rinsing in HBSS. Nonspecific labeling was blocked for 2 h at room temperature using “blocker” solution (2–10% normal goat serum + 5% bovine serum albumin + 1% fish gelatin + 0.1–0.5% Triton X-100 in HBSS. For some experiments 2% normal donkey serum was substituted for normal goat serum). Blocker was removed and a combination of primary antibodies raised in different host species were applied overnight at room temperature. Sections were rinsed in HBSS and then incubated in an appropriate combination of fluorescently conjugated secondary antibodies for 60–75 min at room temperature. Sections were rinsed again and coverslipped using Prolong Gold + DAPI (Life Technologies-Molecular Probes) to retard photobleaching. Specificity of labeling methods was confirmed by omitting primary antibody or substituting normal rabbit serum for primary antibody. Specimens labeled using only one primary antibody and a combination of secondary antibodies showed no bleedthrough of signals between fluorescence channels.

Widefield fluorescence imaging was performed using an Olympus IX70 inverted fluorescence microscope (Olympus America) fitted with a QiCAM CCD camera controlled via QCapture software (QImaging) or, for low magnification imaging, an Olympus MVX10 microscope fitted with an Olympus DP71 camera controlled via CellSens software (Olympus America). Labeling patterns in fluorescence images were assessed by superimposing images of matching fields captured independently in each fluorescence channel. Low magnification image montages were assembled using CellSens or Photoshop software (Adobe Systems). To prepare figures, image scale was calibrated and images were imported into Photoshop software. If necessary, brightness and contrast were adjusted to highlight specific labeling.

### Anatomical identification of brain structures and nomenclature

Identification of structures within the brain, nomenclature, and *in situ* hybridization data for expression of ELOVL4 mRNA was based on the ontological and Nissl-stained anatomic reference atlases for the developing and mature mouse brain from the public resources of the Allen Institute for Brain Science (Lein et al., 2007; Sunkin et al., 2013). Atlases used included: the Allen Developing Mouse Brain Atlas for the E18.5, P4, and P14 mouse brain (Website: 2015 Allen Institute for Brain Science. Allen Developing Mouse Brain Atlas [internet]. Available from: http://developingmouse.brain-map.org); and the Allen Mouse Brain Atlas for the P56 mouse brain (Website: 2015 Allen Institute for Brain Science. Allen Mouse Brain Atlas [internet]. Available from: http://mouse.brain-map.org). For ease of comparison to immunolabeling data, *in situ* hybridization images from the Allen Mouse Brain Atlas for P56 mouse brain were converted from RGB to grayscale and the look-up table was inverted so that cells showing *in situ* hybridization labeling, which originally appeared dark, appeared as bright objects similar to immunofluorescence labeling.

### Antibodies

#### Elongation of very long chain fatty acids 4 (ELOVL4)

Polyclonal rabbit anti-ELOVL4 antibody was generated by our group and the details of its specificity have been published previously (Agbaga et al., 2008; Bennett et al., 2014; Marchette et al., 2014). Anti-ELOVL4 was used at a dilution of 1:300 to 1:500.

#### Glial fibrillary acidic protein (GFAP)

Glial Fibrillary Acidic Protein (GFAP) (Millipore, Cat# MAB360. RRID:AB_2109815; Mouse monoclonal, clone GA-5). Recognizes a band at 51 kDa corresponding to GFAP from human glioma cells (U33CG/343MG) on western blots (Debus et al., 1983) and astrocytes in immunohistochemistry of brain tissue. This antibody does not cross-react with vimentin. Anti-GFAP was used at a dilution of 1:500.

#### Glutamine synthetase (GS)

Glutamine synthetase (GS) (Millipore, Cat# MAB302, RRID:AB_2110656; Mouse monoclonal, clone GS-6). Mouse anti-GS was raised against full length GS purified from sheep brain and recognizes a single 45 kDa band on Western blots of sheep and rat brain (Kentroti et al., 1991). Anti-GS was used at a dilution of 1:500.

#### Neuron-specific nuclear protein (NeuN)

Neuron-specific Nuclear Protein (NeuN) (Millipore, Cat# MAB377B. RRID:AB_177621; Mouse monoclonal, clone A60). Recognizes most postmitotic neurons in the vertebrate brain immunohistochemically and recognizes three bands at 46–48 kDa corresponding to NeuN on western blots (Mullen et al., 1992; Wolf et al., 1996). Anti-NeuN was used at a dilution of 1:500.

All secondary antibodies were conjugated to AlexaFluor tags for visualization by fluorescence microscopy and were used at a dilution of 1:200. Secondary antibodies were Goat anti-rabbit IgG AlexaFluor568 (Cat# A11036; RRID:AB_143011; Molecular Probes), Goat anti-Mouse IgG AlexaFluor488 (Cat# A11029; RRID:AB_138404; Molecular Probes), and Donkey anti-rabbit IgG AlexaFluor568 (Cat# A10042; RRID:AB_2534017; Molecular Probes).

## Results

### Expression and distribution of ELOVL4 in the brain

**Figure 1:**
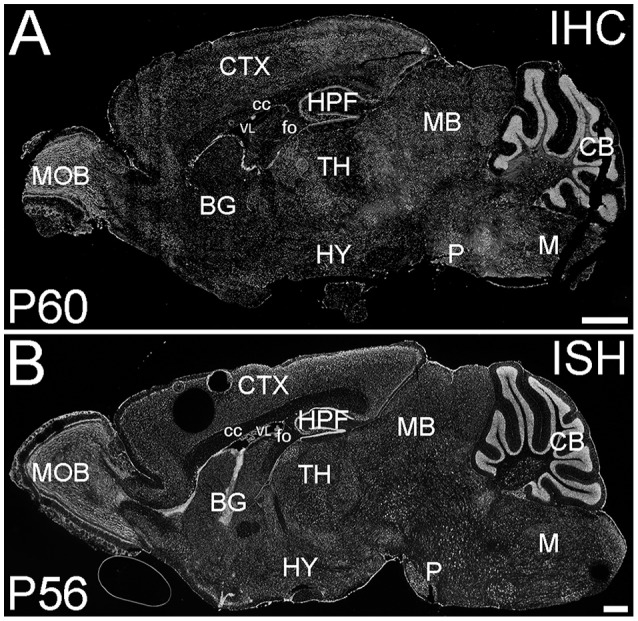
**Distribution of ELOVL4 protein and mRNA in the mouse brain. (A)** Immunohistochemical labeling (IHC) for ELOVL4 in the mouse brain at P60 is widespread, but shows region-specific distribution. **(B)** *In situ* hybridization (ISH) for *Elovl4* mRNA in the mouse brain at P56 also is widespread and shows distribution similar to ELOVL4 protein in **(A)**. (Image from the Allen Institute for Brain Science Allen Mouse Brain Atlas for the P56 mouse brain, image number 69059903_134. http://mouse.brain-map.org). CB, cerebellum; BG, basal ganglia; CTX, cerebral cortex; HPF, hippocampal formation; HY, hypothalamus; M, medulla; MB, midbrain; MOB, main olfactory bulb; P, pons; cc, corpus callosum; fo, fornix; VL, lateral ventricle. Scale bars = 1 mm.

ELOVL4 was expressed widely in the brain at P60; however, its expression was not uniform (Figure 1). ELOVL4 immunolabeling in the brain was prominent in cell bodies, cellular layers, and gray matter nuclei, with less labeling in white matter and little labeling in synaptic layers and neuropil. This labeling pattern closely matched the pattern of *Elovl4* gene expression revealed by *in situ* hybridization (Image from the Allen Institute for Brain Science, Allen Mouse Brain Atlas for the P56 mouse brain, image number 69059903_134. http://mouse.brain-map.org). The pattern of ELOVL4 expression was developmentally regulated, with abundant ELOVL4 labeling present by E18, particularly in neurogenic regions of the brain. The distribution of ELOVL4 immunolabeling in the developing postnatal brain at P10 was transitional between the late embryonic stage and its distribution at by P19–21, which closely resembled that at P60 (see below).

### Distribution of ELOVL4 in specific brain regions

The distribution of ELOVL4-expressing cells in the various regions and nuclei of the brain is described below by region. A more detailed summary is provided in Supplemental Table 1. It is clear that ELOVL4 is expressed by many cell populations throughout the brain. However, the size, number, and complex spatial arrangement of the nuclei and subnuclei and the heterogeneity of cell populations present throughout the brain, did not permit unequivocal identification of all structures and cell types containing ELOVL4-expressing cells. Assessment of developmental patterns of ELOVL4 expression focused on brain regions associated with the CNS-related symptoms (seizures and ataxia) of diseases caused by ELOVL4 mutations: cortex, hippocampal formation, and cerebellum.

#### Telencephalon

##### Cerebral cortex (isocortex)

**Figure 2:**
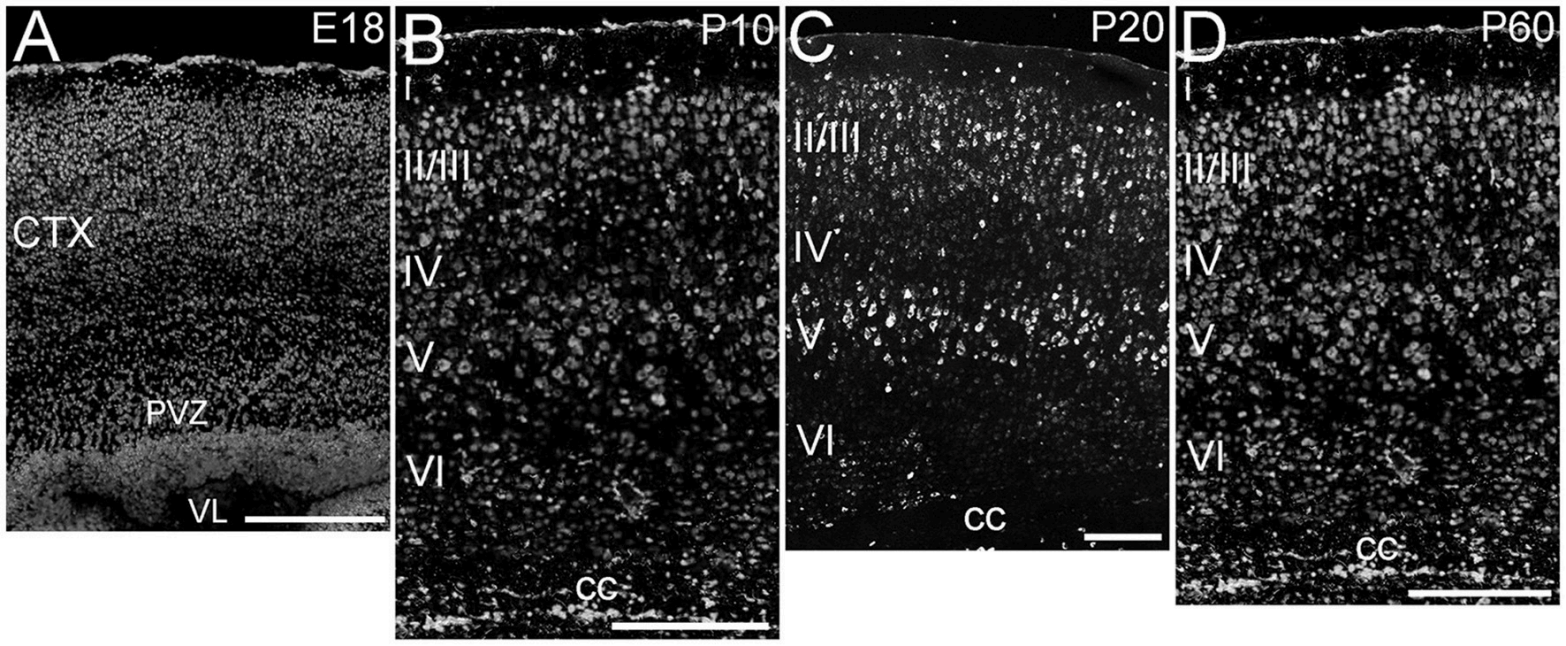
**Expression of ELOVL4 in development of cerebral cortex. (A)** Developing cortex at E18 shows intense ELOVL4 labeling in numerous cells throughout the cortex (CTX). Labeling is especially prominent in the periventricular zone (PVZ), particularly in the region directly adjacent to the lateral ventricle (VL). **(B)** Developing cortex at P10 shows strong labeling of cells in all layers, but the most intensely labeled cells are present in layers II/III and V. Numerous ELOVL4-positive cells also are present in the developing corpus callosum (cc). **(C)** Widespread, layered expression of ELOVL4 is preserved in the cortex at P20. **(D)** The characteristic layered pattern of ELOVL4 expression in cortex is maintained at P60. Scale bars = 250 μm.

ELOVL4 was expressed throughout the cerebral cortex, although labeling intensity varied somewhat among different regions (Figure 2). ELOVL4 labeling was present by E18 and persisted thereafter. At E18, many strongly labeled cells were present in high density in the periventricular zone adjacent to the lateral ventricle and throughout the developing cortex. Some layering of ELOVL4 labeling was present at this stage, but was incomplete. By P10, the cortex showed its characteristic six-layered structure, and the layer-specific pattern of ELOVL4 labeling was present. ELOVL4 labeling was most prominent in the output layers, layers II/III, V, and VI. Labeling for ELOVL4 also was present in layer IV, which receives extensive thalamic inputs and is especially prominent in sensory cortices, but was much less prominent than in layers II/III and V. The sparsely distributed cells in layer I also showed ELOVL4 labeling. Non-sensory areas of cerebral cortex, which have a reduced layer IV, showed less pronounced laminar differences in ELOVL4 labeling. These ELOVL4 labeling patterns persisted at P20 and P60.

##### Olfactory areas

**Figure 3:**
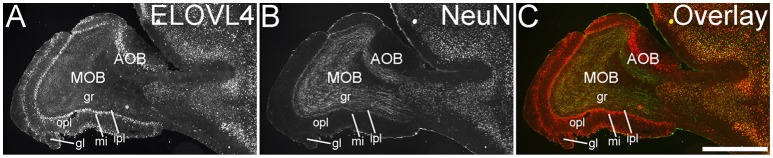
**Expression of ELOVL4 in the olfactory bulb. (A)** Labeling for ELOVL4 is present in the main olfactory bulb (MOB) and accessory olfactory bulb (AOB). Mitral cells in the mitral cell layer (mi) of the MOB and the AOB show intense labeling. Cells surrounding the olfactory glomeruli (gl) and granule cells (gr) also show ELOVL4 labeling. Little labeling is present in the outer plexiform layer (opl) or inner plexiform layer (ipl). **(B)** Labeling for the neuronal marker, NeuN. **(C)** Overlay of **(A,B)**. Scale bar = 1 mm.

The olfactory areas of the telencephalon showed distinct, region-specific ELOVL4 labeling (Figure 3). In the main olfactory bulb, cells surrounding the glomeruli and in the mitral cell layer showed strong ELOVL4 labeling. Cells in the granule cell layer showed less intense ELOVL4 labeling. The accessory olfactory bulb showed a similar pattern of layered ELOVL4 labeling, with intensely labeled cells in the mitral cell layer. Other parts of the olfactory areas also showed ELOVL4 labeling (see Supplementary Table 1). Anterior olfactory nucleus showed many ELOVL4-positive cells, especially in layer 2. Taenia tecta also contained ELOVL4 positive cells. Piriform cortex showed many moderate to strongly labeled cells in the pyramidal layer, with less labeling in the molecular and polymorph layers. The nucleus of the lateral olfactory tract showed numerous cells with weak to moderate ELOVL4 labeling.

##### Hippocampal formation

**Figure 4:**
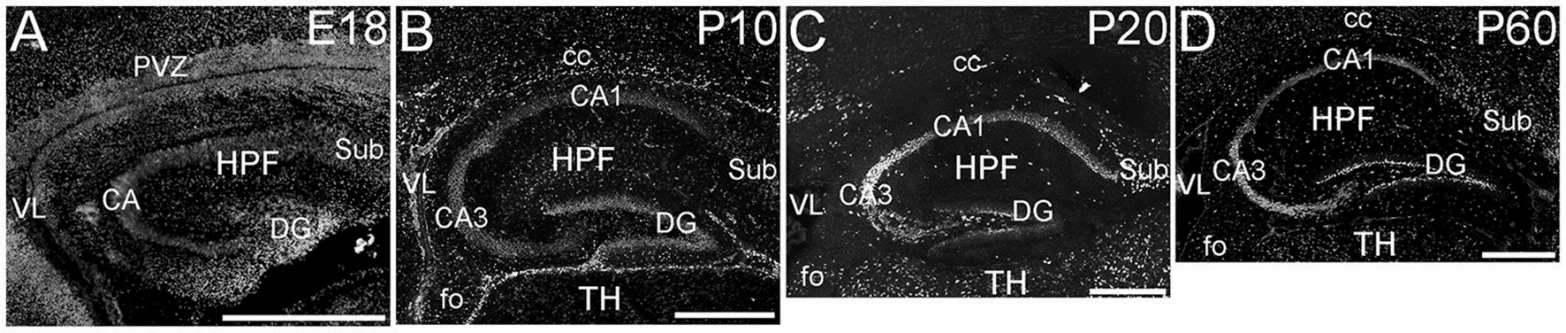
**Expression of ELOVL4 in development of the hippocampal formation. (A)** The developing hippocampal formation (HPF) at E18 shows strong labeling for ELOVL4 in cells of the developing Cornu Ammonis (CA), dentate gyrus (DG), and subiculum (Sub). Intense ELOVL4 labeling is seen near the margins of the lateral ventricle (VL). The periventricular zone (PVZ) of the overlying and developing cerebral cortex iare visible above the hippocampal formation. **(B)**. Developing hippocampal formation at P10, with a well-developed CA region and dentate gyrus. Cells in the CA region show ELOVL4 labeling, with cells in CA3 showing stronger labeling than cells in CA1. Cells in the dentate gyrus and subiculum also show labeling. Labeled cells are also present in the developing corpus callosum (cc) and fornix (fo). Developing thalamus (TH) is visible below the hippocampal formation. **(C)** Hippocampal formation at P20. ELOVL4 labeling is present throughout the CA region, with cells in CA3 showing the strongest labeling. Labeling for ELOVL4 in the granule cell layer of the dentate gyrus is reduced compared to earlier stages although ELOVL4-positive cells persist along the inner margin of the dentate gyrus. Strong labeling is also present in interneurons in the polymorph layer of the dentate gyrus and in the subiculum. **(D)** The hippocampal formation at P60 shows distribution of ELOVL4 labeling similar to that at P20. Scale bars = 500 μm.

The hippocampal formation showed a very distinctive pattern of ELOVL4 expression (Figure 4). At E18, ELOVL4 labeling was present in the cells of the developing Cornu Ammonis (CA) region and the Dentate Gyrus (DG), and subiculum. Many ELOVL4-positive cells also were present along the margin of the hippocampal formation adjacent to the lateral ventricle. By P10, well-developed CA and DG regions had been established. At this stage, ELOVL4 was present in cells throughout the CA region, with slightly stronger ELOVL4 labeling in the CA3 region compared to CA1 and CA2. Cells throughout the DG showed ELOVL4 labeling at this stage. By P20, the difference in ELOVL4 labeling intensity between cells in CA3 and those in CA1 and CA2 was more pronounced. ELOVL4 labeling in interneurons in the polymorphic hilar region (between the blades) of the DG became more prominent. In the DG, ELOVL4 labeling was reduced except in a set of cells lining the inner margin of the DG. The hippocampal formation showed a similar labeling pattern at P60. Labeling for ELOVL4 had a similar distribution in dorsal and ventral hippocampal regions, although labeling tended to be stronger in the dorsal hippocampus. Many cells in presubiculum and postsubiculum also showed strong ELOVL4 labeling (see Supplementary Table 1).

##### Cortical subplate

Cells in the cortical subplate displayed ELOVL4 labeling. Cells in the innermost portion of layer 6 of the isocortex (layer 6b) showed weak to moderate ELOVL4 labeling. Strongly labeled cells located at the border of layer 6b and the corpus callosum were observed in some specimens. The claustrum and the endopiriform nucleus both contained cells showing moderate ELOVL4 labeling (see Supplementary Table 1).

##### Basal ganglia (corpus striatum)

**Figure 5:**
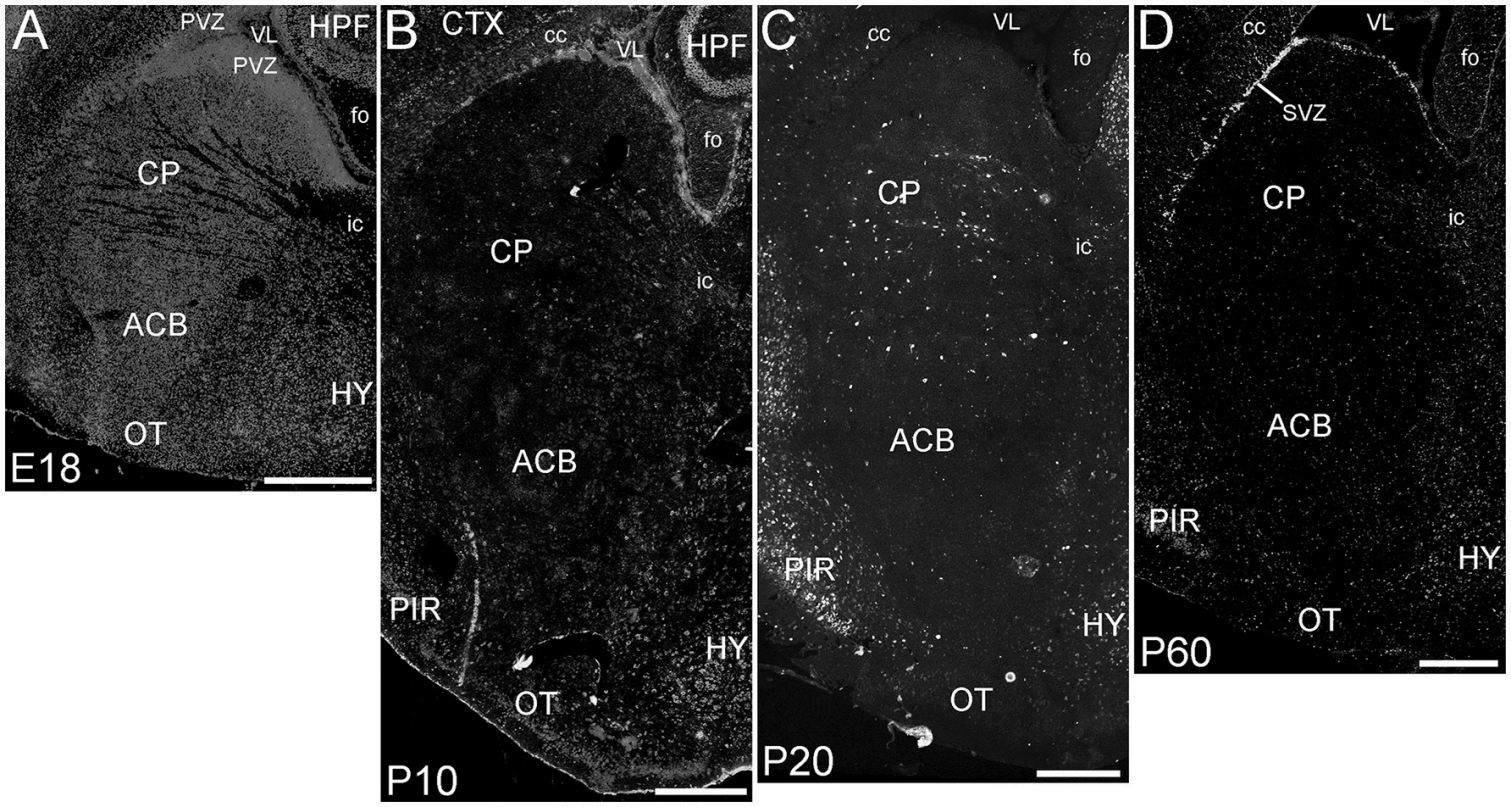
**Expression of ELOVL4 in development of the basal ganglia. (A)** The developing basal ganglia at E18 show very high levels of ELOVL4 expression, particularly in the periventricular zone (PVZ) adjacent to the lateral ventricle (VL). Caudoputamen (CP), nucleus accumbens (ACB), and olfactory tubercle (OT) are shown. **(B)**. Labeling for ELOVL4 in the basal ganglia decreases substantially by P10. **(C)** The basal ganglia maintain low levels of ELOVL4 expression at P20. **(D)** At P60, the basal ganglia maintain relatively low levels of ELOVL4 with sparsely distributed ELOVL4-labeled cells. Labeling for ELOVL4 is also evident in the subventricular zone (SVZ). HPF, hippocampal formation; HY, hypothalamus; PIR, piriform cortex; cc, corpus callosum; fo, fornix; ic, internal capsule. Scale bars = 500 μm.

The basal ganglia (BG) showed a striking pattern of developmental regulation in the expression of ELOVL4 (Figure 5). The developing BG showed higher levels of ELOVL4 labeling than any other part of the brain at E18. Strongly labeled cells were found throughout the BG at this stage, with extremely strong labeling present in the periventricular zone adjacent to lateral ventricles. By P10, ELOVL4 labeling in the BG decreased substantially to levels lower than other regions of the brain, although ELOVL4-positive cells were frequently observed in the subventricular zone underlying the lateral ventricles. This pattern of ELOVL4 labeling was maintained at P20 and P60. This pattern of low ELOVL4 labeling characterized most of the basal ganglia, although the lateral and medial septal nuclei showed somewhat stronger labeling (Supplementary Table 1).

##### Amygdala complex

**Figure 6:**
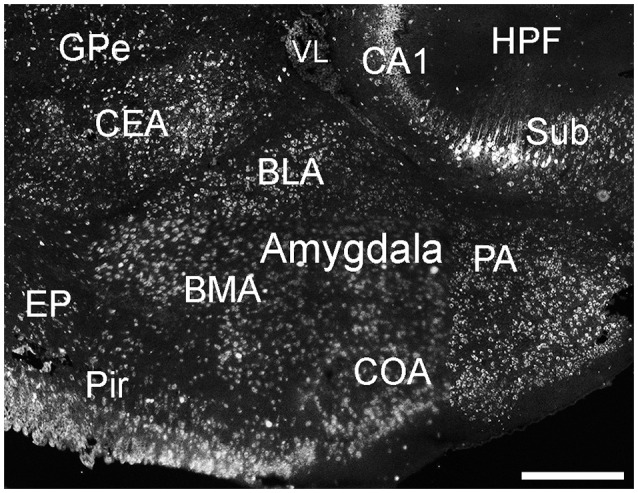
**Expression of ELOVL4 in the amygdala**. Labeling for ELOVL4 is present in the amygdala, but varies among subnuclei. Prominent labeling in the central amygdalar nucleus (CEA), basolateral amygdalar (BLA), basomedial amygdalar nucleus (BMA), posterior amygdalar nucleus (PA), and the cortical amygdalar area (COA) is shown. CA1, Cornu Ammonis, field 1; EP, endopiriform nucleus; GPe, globus pallidus external; GPi, globus pallidus internal; HPF, Hippocampal formation; Pir, piriform cortex; Sub, subiculum; VL, lateral ventricle. Scale bar = 500 μm.

Cells labeled for ELOVL4 were present in many regions of the amygdala complex (Figure 6). The cortical amygdalar area, which is derived from the cortical plate, showed many moderately labeled cells. Cells in the basolateral and basomedial amygdalar nuclei and posterior amygdalar nucleus, which all derive from the cortical subplate, showed moderate ELOVL4 labeling levels. Neurons in the anterior amygdalar area, the central amygdalar nucleus, and the medial amygdalar nucleus, which are all derived from the striatum, also showed ELOVL4-positive cells. Additional ELOVL4-positive cells also were present in other regions of the amygdala, although precise identity of the amygdalar subnuclei containing these cells remains uncertain.

#### Cerebellum

**Figure 7:**
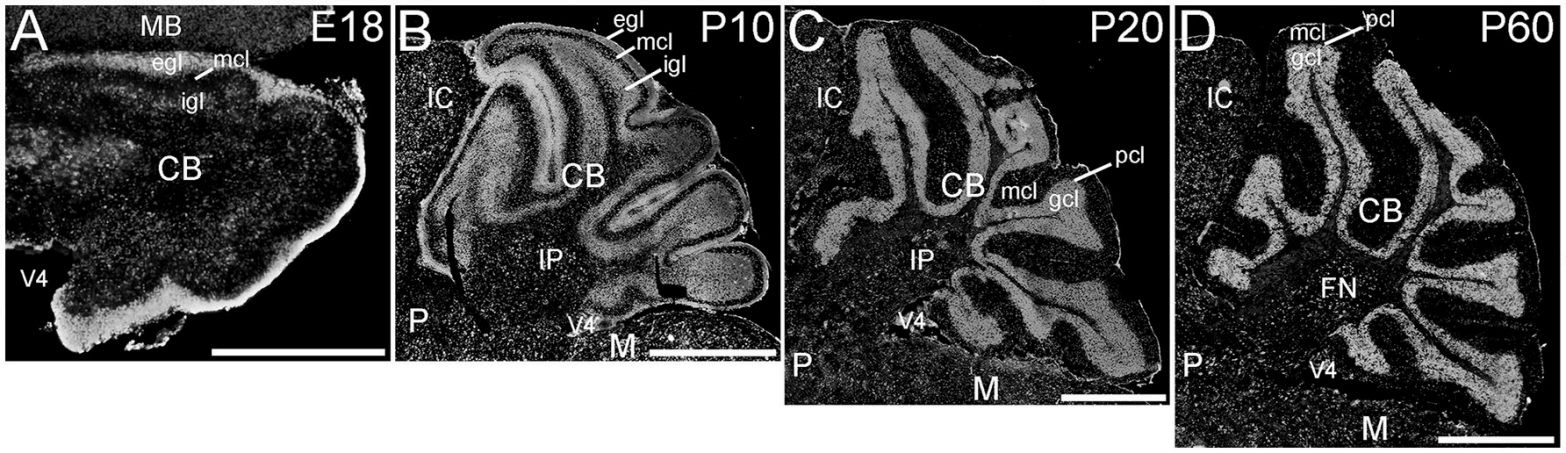
**Expression of ELOVL4 in the developing cerebellum. (A)** ELOVL4 labeling is present in the cerebellum (CB) during early development at E18. Intense labeling is present in the nascent external granule cell layer (egl) of the developing cerebellar cortex. Labeling for ELOVL4 is also present in the developing inner granule cell layer (igl). A few labeled cells also are present in the forming molecular cell layer (mcl). **(B)** Intense ELOVL4 labeling is present in the egl and igl of the cerebellar cortex at P10. Many cells traversing the mcl show ELOVL4 labeling. Strong ELOVL4 labeling is present in the deep cerebellar nuclei by P10 (interpositus nucleus, IP, shown). **(C)** Mature layering of cerebellar cortex is achieved by P20, and is characterized by very intense ELOVL4 labeling in cells in the granule cell layer (gcl). Purkinje cells in the Purkinje cell layer (pcl) also show ELOVL4 labeling, but are difficult to discern at this magnification. The sparsely distributed cells in the mcl also show labeling. Labeling of cells in the deep cerebellar nuclei also persists. **(D)** Distribution of ELOVL4 in the P60 cerebellar cortex and deep cerebellar nuclei (fastigial nucleus, FN, shown) is similar to that in the P20 cerebellum. IC, inferior colliculus; M, medulla; MB, midbrain; P, pons; V4, 4th ventricle. Scale bars = 500 μm for **(A)**; 1 mm for **(B–D)**.

The cerebellum showed striking ELOVL4 labeling at all developmental stages (Figure 7). In the early developing cerebellum at E18, cells in the external granule cell layer showed very strong ELOVL4 labeling. Cells in the inner granule cell layer also showed labeling for ELOVL4, although it was not as strong as that in the external granule cell layer. Cells in the nascent molecular cell layer also showed ELOVL4 labeling. At P10, strong ELOVL4 labeling persisted in the well-defined external granule cell layer, and many ELOVL4 positive cells were present in the molecular cell layer. At this stage, the inner granule cell layer had enlarged substantially and showed increased ELOVL4 labeling. ELOVL4-positive cells also were present in the deep cerebellar nuclei. By P20, cerebellar organization had achieved mature organization with a three-layered cortex comprised of an outer molecular layer, a monolayer of Purkinje cells in the Purkinje cell layer, a thick underlying granule cell layer, and a core of white matter (the arbor vitae). Neurons in the molecular layer showed strong ELOVL4 labeling. In contrast, Purkinje cells showed only moderate levels of ELOVL4 labeling. Neurons in the granule cell layer, however, showed the most intense ELOVL4 labeling of any cells in the entire brain. All parts of cerebellar cortex showed this pattern of ELOVL4 labeling. In addition, many neurons in the deep cerebellar nuclei (fastigial, interposed and dentate nuclei) showed strong labeling for ELOVL4. This pattern of labeling was maintained at P60.

#### Diencephalon

##### Thalamus

**Figure 8:**
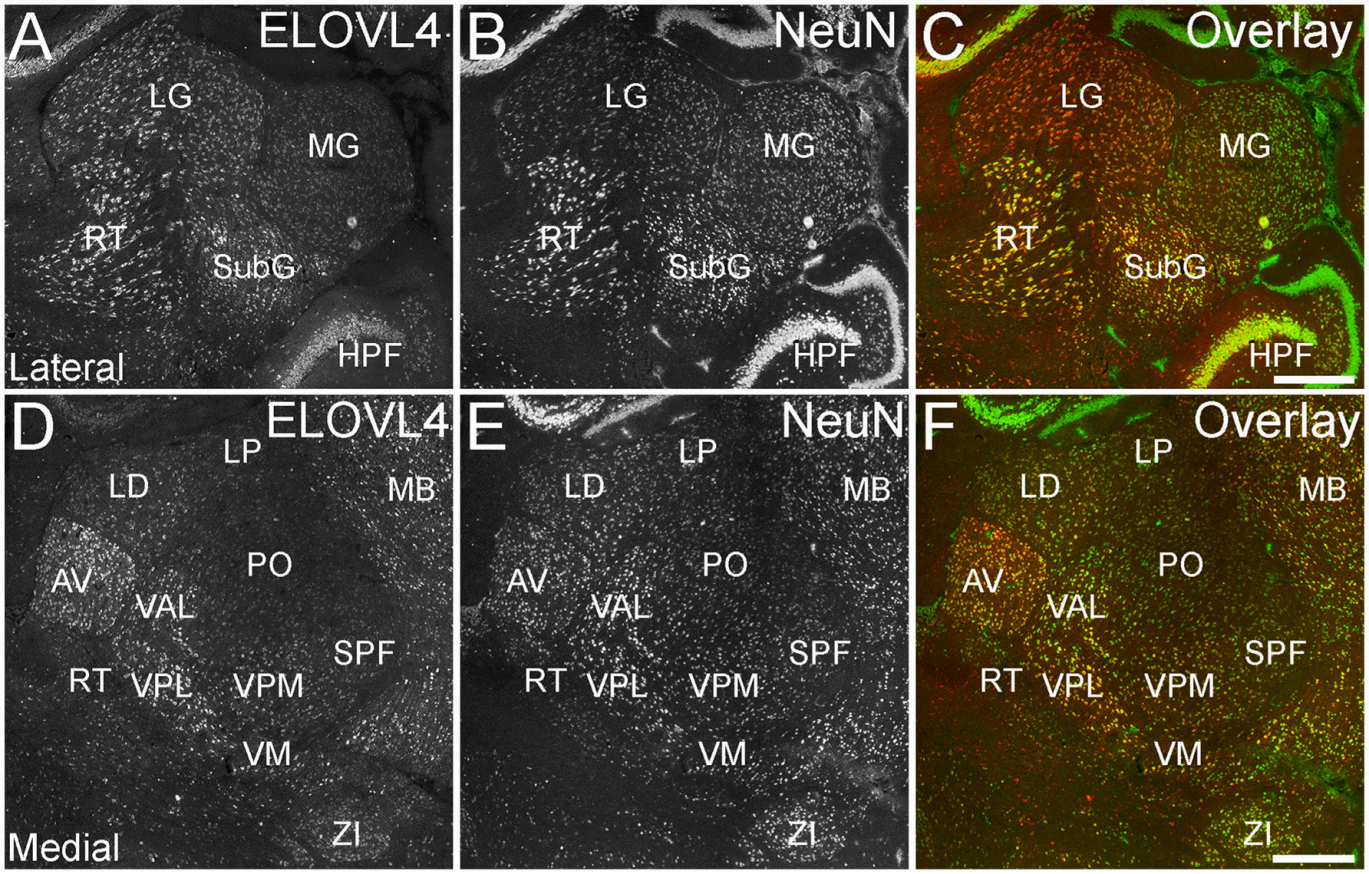
**Expression of ELOVL4 in the thalamus. (A)** Prominent labeling for ELOVL4 in the lateral thalamus is present in the lateral geniculate nucleus (LG), reticular nucleus (RT), and subgeniculate nucleus (SubG). The medial geniculate nucleus (MG) shows much weaker ELOVL4 labeling. **(B)** Labeling for the neuronal marker, NeuN, in lateral thalamus. **(C)** Overlay of **(A,B)**. **(D)** Labeling for ELOVL4 also is present in more medial regions of the thalamus. Strong labeling is present in the anteroventral (AV), ventral posterolateral (VPL), and ventral anterior-lateral complex (VAL) nuclei. Moderate ELOVL4 labeling is present in the lateral dorsal (LD), ventral posteromedial (VPM), and ventromedial (VM) nuclei. Lateral posterior (LP), posterior complex (PO), and subparafascicular (SPF) nuclei of the thalamus show less ELOVL4 labeling. **(E)** Labeling for NeuN in the medial thalamus. **(F)** Overlay of **(D,E)**. HPF, hippocampal formation; MB, midbrain; ZI, zona incerta. Scale bars = 500 μm.

ELOVL4 was expressed broadly in the thalamus, but its expression varied considerably among thalamic nuclei (Figure 8; Supplementary Table 1). A general pattern of ELOVL4 labeling noted in the thalamus was that nuclei located in the anterior portion of the thalamus often showed stronger ELOVL4 labeling than nuclei located in the center or posterior portions of the thalamus. Sensory motor cortex-related nuclei typically contained a number of neurons that showed weak to moderate ELOVL4 labeling, although some of these nuclei showed little labeling. Polymodal-association cortex-related nuclei showed prominent ELOVL4 labeling, with some of these nuclei, particularly in the anterior thalamus, showing very strong labeling. Cells in the reticular nucleus of the thalamus varied in their ELOVL4 labeling from strong to weak. Double labeling with the neuronal marker NeuN showed that ELOVL4 was expressed predominantly by thalamic neurons. Additional neurons in the thalamus also showed ELOVL4 expression, but the identity of the nuclei containing these cells was not conclusive.

##### Hypothalamus

**Figure 9:**
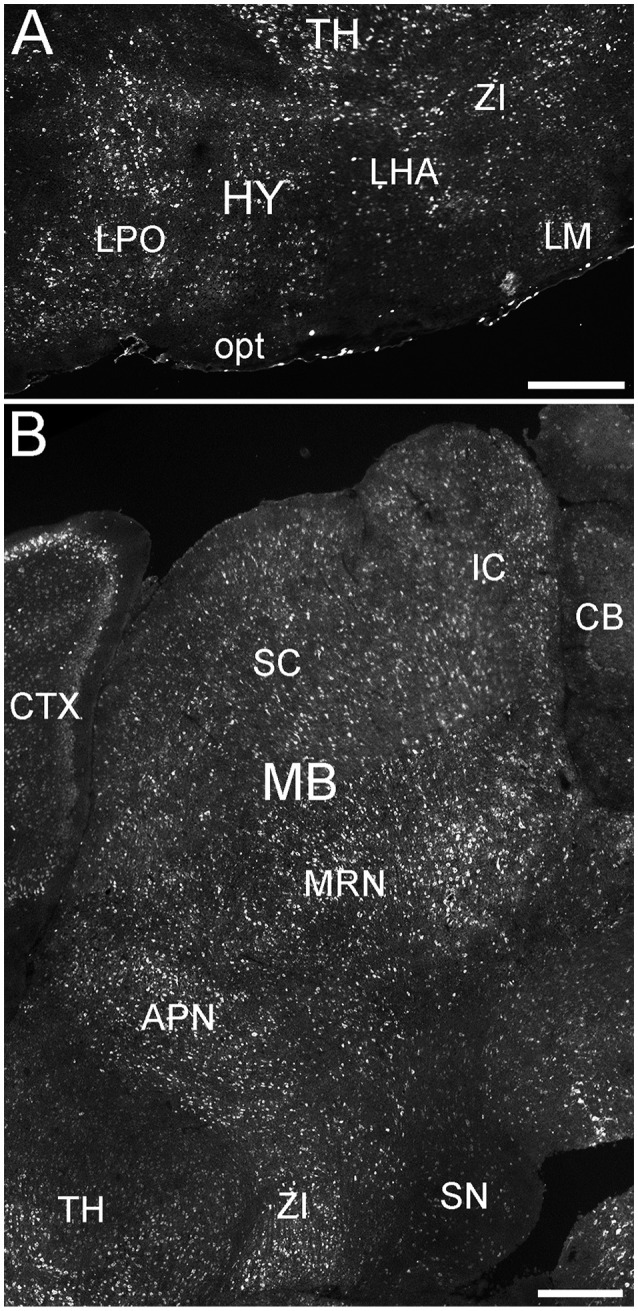
**Expression of ELOVL4 in the hypothalamus and midbrain. (A)** Extensive ELOVL4 labeling is present in the hypothalamus (HY). Prominent labeling is seen in the lateral preoptic area (LPO), lateral mammilary nucleus (LM), zona incerta (ZI), and in parts of the lateral hypothalamic area (LHA). TH, Thalamus; opt, optic tract. **(B)** Labeling for ELOVL4 in the midbrain is widespread but varies among nuclei. Prominent labeling is present in the superior colliculus (SC), inferior colliculus (IC), midbrain reticular nucleus (MRN), and anterior pretectal nucleus (APN). Substantia nigra (SN) shows much less labeling for ELOVL4 than other portions of the midbrain. Cerebellum (CB), cortex (CTX), and zona incerta (ZI) also are visible. Scale bars = 500 μm.

Many parts of the hypothalamus contained ELOVL4-positive neurons, although labeling was not uniform throughout the region (Figure 9A; Supplementary Table 1). The anterior nucleus and the mammillary body, parts of the hypothalamic medial zone-behavior control column, showed weak to moderately labeled cells. The hypothalamic lateral zone, the lateral hypothalamic area, lateral preoptic area, subthalamic nucleus, and zona incerta all contained moderately to strongly labeled cells. Additional ELOVL4-positive cells were present in other parts of this zone. ELOVL4-positive cells also were present in the periventricular region and the periventricular zone-neuroendocrine motor zone, although the precise identity of the associated nuclei was not conclusive.

#### Mesencephalon (midbrain)

ELOVL4 labeled cells were present in many parts of the midbrain (Figure 9B; Supplementary Table 1). The superior and inferior colliculi contained numerous ELOVL4-positive cells, with additional ELOVL4-positive cells present in other portions of the midbrain sensory related nuclei. ELOVL4 labeling varied widely among motor related nuclei in the midbrain. The motor portion of the superior colliculus, midbrain reticular nucleus, periaqueductal gray, pretectal region, and red nucleus all showed varying levels of ELOVL4 labeling and numbers of ELOVL4-positive cells. Notably, substantia nigra and the ventral tegmental area showed little ELOVL4 labeling.

#### Rhombencephalon

##### Metencephalon (pons)

**Figure 10:**
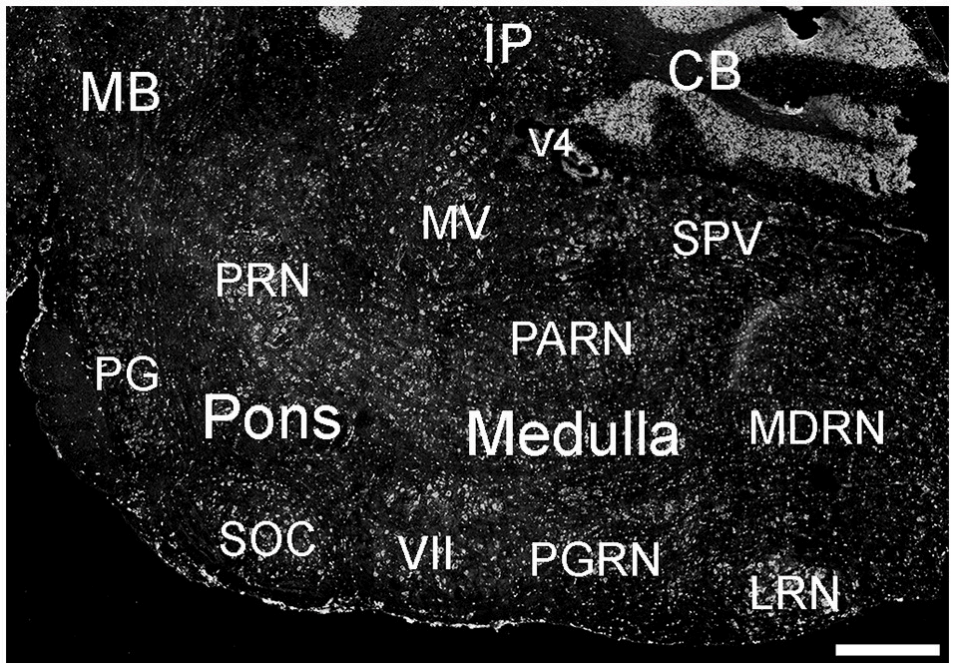
**Expression of ELOVL4 in the pons and medulla**. Many labeled cells are present in the pons, including cells in the pontine reticular nucleus (PRN), pontine gray (PG), and superior olivary complex (SOC). In the medulla, ELOVL4-positive cells are prominent in the medial vestibular nucleus (MV), facial motor nucleus (VII), spinal nucleus of the trigeminal (SPV), parvicellular reticular nucleus (PARN), medullary reticular nucleus (MDRN), and later reticular nucleus (LRN). CB, cerebellum; IP, interpositus nucleus of the cerebellum; MB, midbrain; V4, fourth ventricle. Scale bar = 500 μm.

Many parts of the pons contained ELOVL4-positive cells (Figure 10; Supplementary Table 1). Sensory related nuclei showing prominent ELOVL4 labeling included the principal sensory nucleus of the trigeminal, parabrachial nucleus, and superior olivary complex. Several motor related nuclei showed moderate to strong labeling for ELOVL4 including, the pontine central gray, pontine gray, the caudal portion of the pontine reticular nucleus and the motor nucleus of the trigeminal. The behavioral state related rostral portion of the pontine reticular nucleus showed weak to moderate labeling. Additional ELOVL4-positive neurons were also present in the pons, but the specific identity of the nuclei they were associated with was uncertain.

##### Myencephalon (medulla)

Many areas within the medulla showed labeling for ELOVL4 (Figure 10; Supplementary Table 1). Sensory related nuclei in the medulla with ELOVL4 labeled cells included the dorsal and ventral cochlear nuclei and the spinal nucleus of the trigeminal. Motor related nuclei in the medulla that showed prominent ELOVL4 labeling included the reticular nuclei and vestibular nuclei. Additional ELOVL4-positive neurons also were noted in areas of the medulla likely to be associated with sensory, motor and behavioral functions, but identification of specific nuclei containing these cells was not conclusive.

### ELOVL4 is expressed primarily by neurons

**Figure 11:**
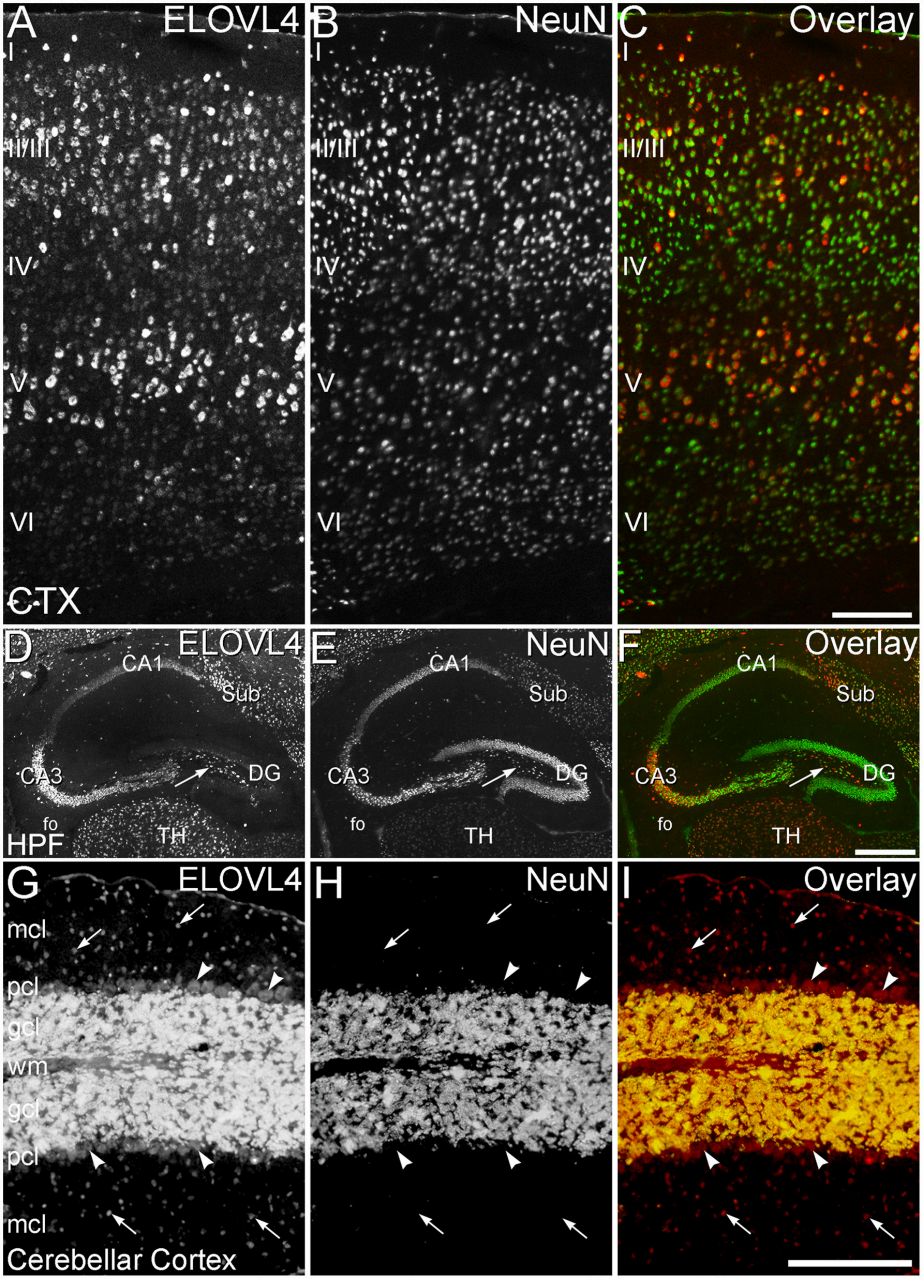
**ELOVL4 expression is primarily neuronal. (A–C)** Cerebral Cortex (CTX). **(A)** Labeling for ELOVL4 is present in all layers of the cerebral cortex (CTX). Cells in the pyramidal layers (II/III and V) are most prominently labeled, but ELOVL4-positive cells also are present in the molecular layer (I), layer 4 (IV), and layer 6 (VI). **(B)** Labeling for the neuronal marker, NeuN. **(C)** Overlay of panels **(A,B)** shows close correspondence of ELOVL4 (red) and NeuN labeling (green), indicating that ELOVL4 is primarily expressed by neurons in the cortex. **(D–F)** Hippocampal formation. **(D)** Labeling for ELOVL4 is present in the cellular layers of the hippocampal formation (HPF), including the Cornu Ammonis, with field 1 (CA1) showing less prominent labeling than field 3 (CA3). Prominent ELOVL4 labeling also is present in the subiculum (sub) and interneurons in the polymorph layer (arrow). Cells along the inner margin of the dentate gyrus (DG) show moderate ELOVL4 labeling, but most dentate granule cells show little ELOVL4 labeling. **(E)** Labeling for the neuronal marker, NeuN. **(F)** Overlay of **(D,E)** shows close correspondence of ELOVL4 (red) and NeuN (green) labeling, indicating that ELOVL4 is primarily expressed by neurons in the hippocampal formation. TH, Thalamus. **(G–I)** Cerebellar cortex. **(G)** Cross section through a cerebellar folium showing ELOVL4 expression in the cerebellar cortex. Neurons (arrows) in the molecular cell layer (mcl) show strong ELOVL4 labeling, but the monolayer of large Purkinje cells (arrowheads) that form the Purkinje cell layer (pcl) shows only moderate levels of ELOVL4 labeling. The densely packed cells of the granule cell layer (gcl) show very intense labeling. **(H)** Labeling for the neuronal marker, NeuN, strongly labels neurons in the gcl, but not Purkinje cells, as appropriate. **(I)** Overlay of panels **(G,H)** shows close correspondence of intense ELOVL4 (red) and NeuN (green) labeling resulting in an orange color in the gcl. These results indicate that ELOVL4 in the cerebellum is primarily expressed by neurons. wm, white matter of the arbor vitae. Scale bars = 200 μm for **(A–C,G–I)**; 500 μm for **(D–F)**.

To better understand the potential roles of ELOVL4 and its VLC-FA products in the brain, double immunolabeling for ELOVL4 in conjunction with known neuronal and glial markers was used to identify the types of cells that expressed ELOVL4. To assess ELOVL4 expression in neurons, double labeling for ELOVL4 and NeuN, a widely expressed neuron-specific transcription factor, was performed. Labeling for ELOVL4 and NeuN showed nearly identical distributions in most brain regions, indicating that the expression of ELOVL4 was primarily neuronal (Figure 11, cerebral cortex, hippocampal formation, and cerebellar cortex shown. Also see Figures 3, 8, olfactory bulb and thalamus, respectively). It is important to note that ELOVL4 expression by neurons was not ubiquitious. Specific populations of neurons, including populations of neurons in the BG, parts of the thalamus, and substantia nigra, showed little expression of ELOVL4.

**Figure 12:**
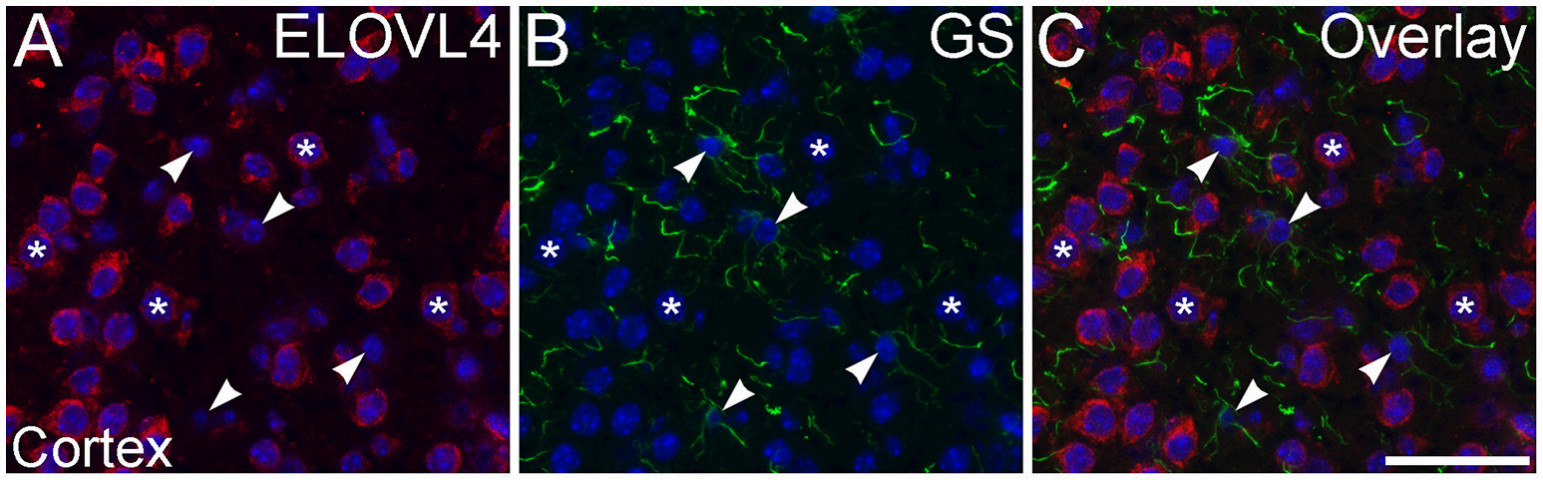
**Astrocytes show little ELOVL4 labeling. (A)** Labeling for ELOVL4 is present in many cells in the cerebral cortex (^*^). **(B)** Astrocytes (arrowheads), identified by labeling for glutamine synthetase (GS). **(C)** Overlay of **(A,B)** shows little labeling for ELOVL4 in astrocytes. Nuclei counterstained with DAPI (blue) in all panels. Scale bar = 50 μm.

**Figure 13:**
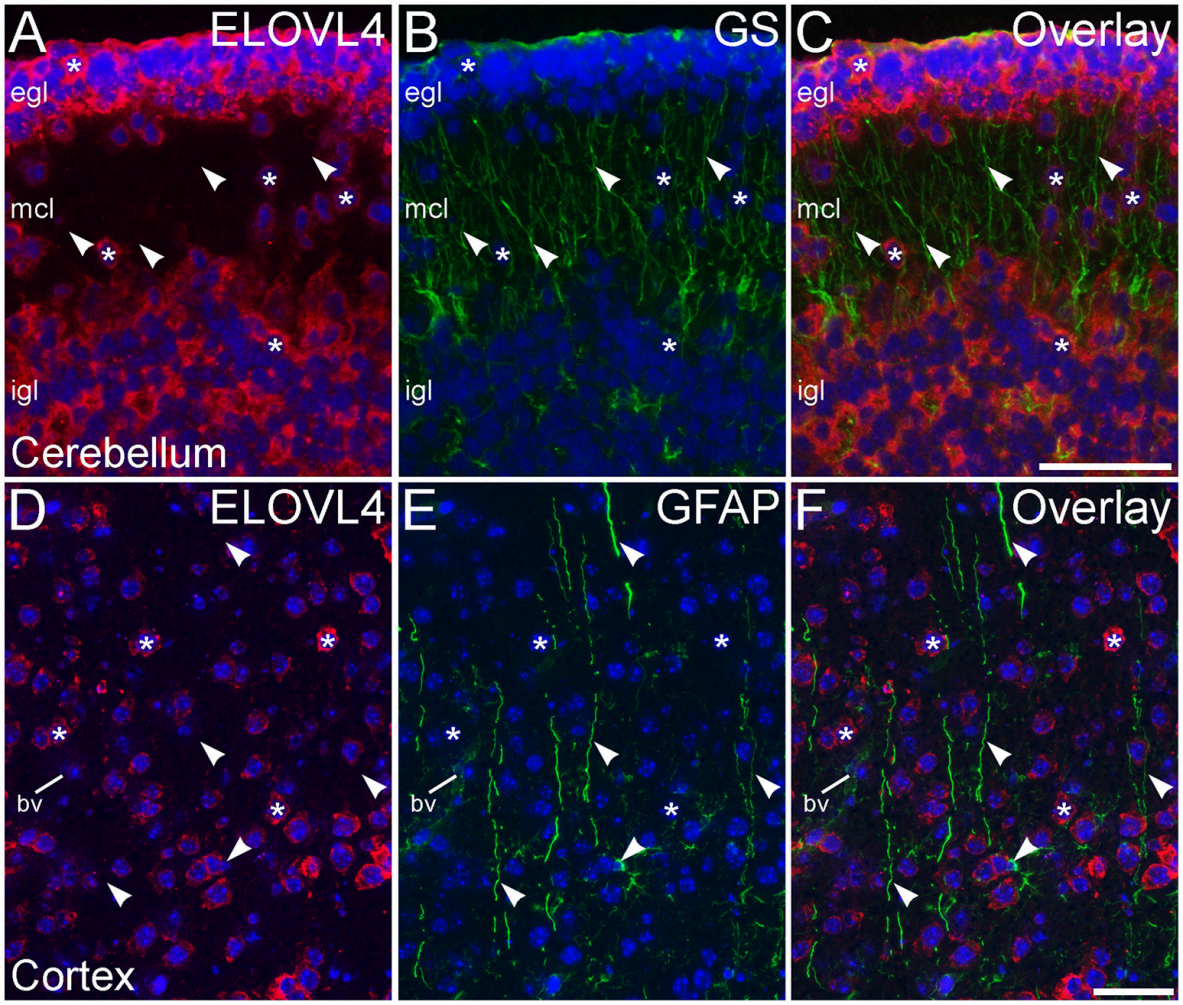
**Radial glial cells show little ELOVL4 labeling. (A)** Many cells (^*^) in the external and internal granule cell layers (egl and igl, respectively), as well as cells migrating through the molecular cell layer (mcl), of the developing cerebellum show ELOVL4 labeling at P10. **(B)** Radial glial cells and their processes (arrowheads) spanning the layers of the cerebellum can be identified by labeling for glutamine synthtase (GS). **(C)** Overlay of **(A,B)** shows no colocalization of labeling for ELOVL4 and GS. **(D)** Many cells in the developing cerebral cortex show ELOVL4 labeling (^*^) at P10. **(E)** Radial glial cells and their processes (arrowheads), identified by labeling for glial fibrillary acidic protein (GFAP). Non-specfic labeling of a blood vessel (bv) also is visible. **(F)** Radial glial cells and their processes do not show labeling for ELOVL4. Nuclei counterstained with DAPI (blue) in all panels. Scale bars = 50 μm.

To assess ELOVL4 expression by astrocytes and radial glia, brain sections were immunolabeled for ELOVL4 in conjunction with the astrocyte markers GFAP or GS. Astrocytes showed little or no ELOVL4 labeling (Figure 12). Similarly, radial glial cells, which are important to proper migration of cells during development, did not show ELOVL4 labeling in either the developing cortex or the developing cerebellum at P10 (Figure 13). These cells were identified by labeling for GS or GFAP, and their long, radially oriented processes that projected vertically through the cerebral cortex to the cortical surface or from the cerebellar granule cell layer to the cerebellar surface. In contrast, cells adjacent to the ascending radial glial processes, presumably representing immature migrating neurons, showed strong ELOVL4 labeling.

**Figure 14:**
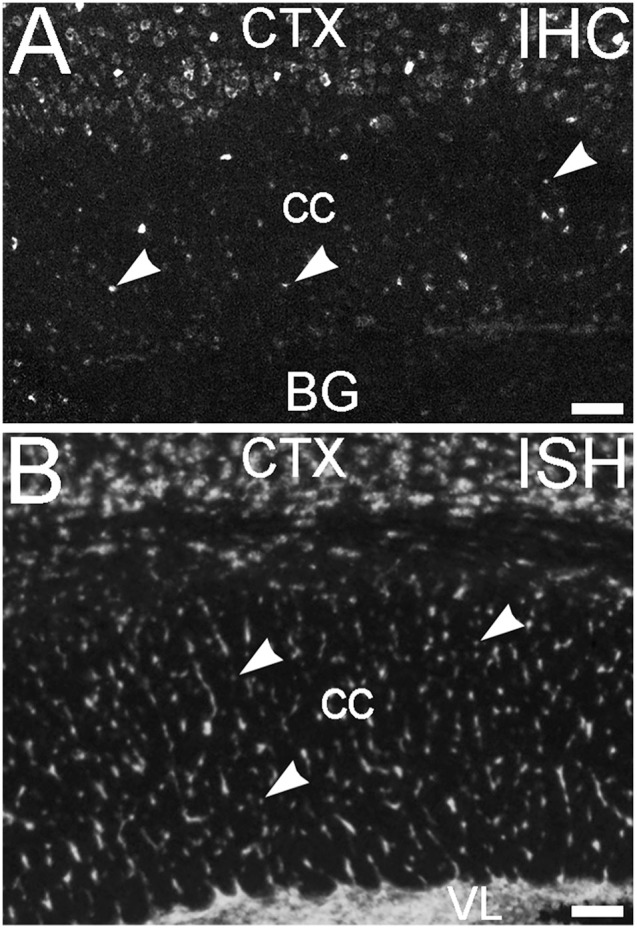
**Glial cells in white matter express ELOVL4. (A)** Immunohistochemical labeling (IHC) for ELOVL4 identifies presumptive oligodendrocytes (arrowheads) in white matter. Corpus callosum (cc) shown. CTX, cerebral cortex; BG, basal ganglia. **(B)** *In situ* hybridization (ISH) for *Elovl4* mRNA also reveals small cells in corpus callosum (arrowheads). VL, lateral ventricle. (Image from the Allen Institute for Brain Science Allen Mouse Brain Atlas for the P56 mouse brain, image number 69059903_134. http://mouse.brain-map.org). Scale bars = 100 μm.

An additional non-neuronal population of cells that showed ELOVL4 labeling was observed in white matter tracts, such as the corpus callosum and the fornix (Figure 14A, corpus callosum shown). *In situ* hybridization for *Elovl4* mRNA confirmed that these cells expressed the *Elovl4* gene (Figure 14B; from Allen Institute for Brain Science, Allen Mouse Brain Atlas for the P56 mouse brain, image number 69059903_134. http://mouse.brain-map.org). Given their small size and placement in white matter, it is likely that these ELOVL4 positive cells represent oligodendrocytes, which are responsible for myelination of axons and are most numerous in white matter, although a small number of stray neurons have been described in the corpus callosum of the mouse by some authors.

## Discussion

The immunolabeling studies presented here are the first to investigate the distribution of ELOVL4, the fatty acid elongase enzyme needed for synthesis of VLC-FAs, in the brain. These studies show that ELOVL4 is widely expressed throughout the brain, consistent with important roles for ELOVL4 and its VLC-FA products in brain function. Importantly, ELOVL4 expression is not uniform, even within a given brain structure, suggesting that ELOVL4 and VLC-FA function varies in a region- and cell type-specific manner. Comparison of ELOVL4 expression patterns in the brain from late embryonic stages to mature stages also shows region-specific developmental regulation of ELOVL4, further supporting the notion that ELOVL4 and VLC-SFA function is differentially regulated throughout the brain. Together, these results characterize the highly specific patterns of ELOVL4 expression in the developing and mature brain and characterize anatomical details of ELOVL4 expression in a number of functionally important parts of the brain. However, this characterization is not complete and many important ELOVL4-containing structures, nuclei, and cell populations remain to be identified.

### Cell-specific expression of ELOVL4 in the brain

An important finding is that ELOVL4 expression and, therefore, VLC-SFA synthesis, is mainly associated with neurons. This suggests that disease and symptoms associated with mutations in ELOVL4 including neurodegeneration, seizures, intellectual disability, and ataxia, arise primarily from neuronal, rather than glial, dysfunction. It is also clear that neuronal expression of ELOVL4 is not strictly related to any specific neurotransmitter phenotype, or specifically associated with excitatory or inhibitory neurotransmission. Many glutamatergic cells, such as CA3 pyramidal cells in hippocampus, cortical pyramidal cells, and cerebellar granule cells show strong labeling for ELOVL4. In contrast, other glutamatergic cells, such as granule cells in the mature dentate gyrus of the hippocampus, show little labeling for ELOVL4. Cerebellar Purkinje cells, which utilize GABA as a neurotransmitter, also express ELOVL4, indicating that ELOVL4 expression is not limited to glutamatergic cell types. In contrast, the striatum, which contains many GABAergic neurons, shows very little ELOVL4 labeling. Similarly, the substantia nigra, which contains large numbers of dopaminergic neurons shows little ELOVL4 labeling. Thus, ELOVL4 and the function of it's product VLC-fatty acids is likely neuron- or synapse-specific.

Another important finding in these immunolabeling studies is that some oligodendrocytes were labeled, but not all. *In situ* hybridization data support this notion. The functional role of ELOVL4 and VLC-SFAs in oligodendrocytes which are responsible for myelination of axons and are found mainly in white matter tracts, and myelin has not been studied to date.

Immunolabeling studies show that ELOVL4 was not expressed to any appreciable extent by astrocytes or radial glia. Thus, VLC-SFAs are unlikely to have major functions in these cells. An important caveat is the growing recognition of the neurochemical diversity of astroglia, which have been suggested recently to possess diversity rivaling that of neurons (Ben Haim and Rowitch, 2017). Neither GFAP nor GS are expressed by all astrocytes. Therefore, it is possible that populations of astrocytes that do express ELOVL4 could exist, but were not identified in the current studies. However, the preponderance of ELOVL4 expression by neurons, suggests that any role for ELOVL4 and VLC-SFA in astrocytes would likely be limited. Transient expression of ELOVL4 by astrocytes or radial glia potentially could exist, and might not have been detected in our experiments due to the sampling intervals in our studies.

ELOVL4 localized to cell bodies, consistent with its previous identification as an endoplasmic reticulum protein (Ambasudhan et al., 2004; Karan et al., 2004; Grayson and Molday, 2005); however, the available data indicate that its VLC-FA products are localized to structures removed from the soma. In photoreceptors, VLC-PUFA are localized to the membranes of the light sensitive outer segments (Aveldano, 1987; Aveldano and Sprecher, 1987; Agbaga et al., 2008). Mechanisms for such selective sorting of VLC-FAs are unknown, but specific mechanisms to move lipids selectively among membrane compartments have been described for other lipid classes (Jackson et al., 2016). Alternatively, VLC-FAs might be transported to the specific subcellular compartments via directed vesicular trafficking. Similarly, ELOVL4 localization to presumptive oligodendrocyte cell bodies suggests VLC-FAs are synthesized in the soma. The mechanisms underlying the trafficking and sorting of VLC-FAs is an important unresolved issue.

### Potential roles for ELOVL4 and VLC-SFAs in development

The developmental regulation of ELOVL4 expression suggests that VLC-SFAs may have important, temporally regulated roles in brain development. The neurodevelopmental deficits (intellectual disability, seizures) observed in patients with homozygous inheritance of mutant forms of ELOVL4 support this notion (Aldahmesh et al., 2011; Mir et al., 2014). However, these functions are currently unknown and whether VLC-FA profiles in specific regions of the brain change over the course of development is also unknown.

The developmental patterns of ELOVL4 expression suggest potential roles for VLC-SFAs in cell proliferation, migration, and/or differentiation. Labeling for ELOVL4 is very prominent in cells in proliferative regions of the brain. These regions include, the periventricular zone of the lateral ventricles and the rhombic lip and external granule cell layer of the developing cerebellum at E18. At postnatal stages, the subventricular zone of the lateral ventricles and the inner margin of the hippocampal dentate gyrus, which house neural progenitor cells into adulthood, also contain cells with distinct ELOVL4 labeling. ELOVL4 levels remained high in migrating cells in the basal ganglia, cerebral cortex, hippocampus, and cerebellum. Thus, ELOVL4 and, presumably, its VLC-FA products are present in individual cells in the brain from very early stages of their differentiation, potentially even prior to cell fate commitment. However, the role of ELOVL4 and VLC-SFAs in cell proliferation, differentiation, and cell migration has not been investigated directly to date.

### Relationship of ELOVL4 and disease

Inheritance of mutant *ELOVL4* alleles has important consequences in the CNS that depend on the specific mutation inherited and whether inheritance is heterozygous or homozygous, indicating that VLC-FAs are critical to normal health and brain function (Bernstein et al., 2001; Edwards et al., 2001; Zhang et al., 2001; Aldahmesh et al., 2011; Cadieux-Dion et al., 2014; Mir et al., 2014; Bourassa et al., 2015; Ozaki et al., 2015; Agbaga, 2016). The relationship of ELOVL4 mutations, inheritance patterns, and symptoms in human disease is complex, and the precise mechanisms underlying the presentation of each disease are unclear (Agbaga, 2016). However, the distribution of ELOVL4 in the brain may provide some insight into the sites in the brain that contribute to specific deficits. The seizures that are characteristic of homozygous inheritance of mutant *ELOVL4* alleles (Aldahmesh et al., 2011; Mir et al., 2014) are consistent with the expression of ELOVL4 in neurons located in hippocampus, thalamus, and the cortex itself, all of which can serve as initiation points for seizures. Homozygous deficits in *ELOVL4* and its VLC-FA products potentially could contribute to intellectual disability, which is associated with anatomical and physiological deficits in the cortex. Homozygous *ELOVL4* deficits in the cerebellum and in oligodendrocytes also might contribute to spasticity and dis-coordination of movement in these diseases. The striking down-regulation of ELOVL4 in the basal ganglia suggests that these regions of the brain are not likely to be the underlying cause of the neural dysfunction in diseases arising from mutant forms of *ELOVL4*. The slower neurodegenerative changes that characterize disease arising from heterozygous inheritance of mutant *ELOVL4* alleles in STDG3 (Bernstein et al., 2001; Edwards et al., 2001; Zhang et al., 2001) and SCA-34 (Cadieux-Dion et al., 2014; Bourassa et al., 2015; Ozaki et al., 2015) may reflect slow compromise of neural function due to the presence of a single dysfunctional *ELOVL4* allele. However, a particularly intriguing question is why one set of *ELOVL4* mutations would lead to a retinal degeneration with no other problems in the brain, while a different set of *ELOVL4* mutations would cause spinocerebellar ataxia without any retinal involvement. One possibility is that these differences arise from different mechanisms regulating *ELOVL4* gene expression in the retina and in the cerebellum. Another interesting possibility is that the substrates and lipid metabolism pathways in the retina and the cerebellum differ in ways that lead to production of VLC-PUFA in the PC fraction in the retina, but leads to production of VLC-SFAs in the cerebellum and elsewhere in brain (Agbaga, 2016).

The precise molecular mechanisms by which ELOVL4 deficiencies disrupt function in the nervous system remain obscure. However, the presence of VLC-FAs in membrane lipids might be expected to reduce membrane fluidity, which could influence trafficking, packing of components within the membrane, membrane stability, or affect the arrangement of microdomains within a membrane. These ideas remain to be tested.

## Author contributions

Study concept and design: DS, BH, FD, MA, RA. Analysis and interpretation of data; Critical revision of the manuscript for important intellectual content; Administrative, technical, and material support: DS, BH, MS, KO, NR, FD, MA, RA. Drafting of the manuscript: DS. Study supervision: DS, RA. All Authors agree to be accountable for all aspects of the studies.

## Funding

The research reported here was supported by: NIH R21 NS090117 and NIH P30 EY021725 to RA; NIH F31 NS089358 to BH; Knight Templar Eye Foundation and BrightFocus Foundation grants to MA.

### Conflict of interest statement

BH, MA, and RA have a United States Patent for use of VLC-FA in treatment of neurodegenerative and skin diseases involving depletion of VLC-FA. The other authors declare that the research was conducted in the absence of any commercial or financial relationships that could be construed as a potential conflict of interest.
