# Supplementary material for: Distribution of ELOVL4 in the Developing and Adult Mouse Brain
Source: Front Neuroanat. 2017 May 1;11:38. doi: 10.3389/fnana.2017.00038 (PMC5410580; doi:10.3389/fnana.2017.00038)
Supplement: Supplementary file 1 [file Table1.docx]

**Supplementary Table 1. Summary of ELOVL4 immunolabeling distribution in the mouse brain.** Table summarizes ELOVL4 distribution in the mouse brain after P20. (-) little or no ELOVL4 labeling; (+) weak ELOVL4 labeling; (++) moderate ELOVL4 labeling; (+++) strong ELOVL4 labeling; (++++) intense ELOVL4 labeling. (-/+) nucleus contains combination of ELOVL4 immuno-negative cells and weakly ELOVL4-positive cells.

| **Brain Region** | **Area or Nucleus** | **ELOVL4 Labeling** | **Notes** |
| --- | --- | --- | --- |
| **TELENCEPHALON** |  |  |  |
| Cerebral cortex (CTX) |  |  |  |
| Cortical plate (CTXpl) |  |  |  |
| Isocortex (ISO) | Frontal pole (FRP) | ++/+++ | Numerous labeled cells, with even distribution across layers. |
|  | Somatomotor areas (MO) | ++/+++ | Many labeled cells present. Most moderately labeled, but stronger in layer 5. |
|  | Somatosensory areas (SS) | +/++/+++ | Relatively few labeled cells in layers 1 and 4; ++ in layer 2/3, 6a; +++ in layer 5. |
|  | Infralimbic area (ILA) | ++/+++ | Many labeled cells present. Most moderately labeled, but stronger in layer 5. |
|  | Visual areas (VIS) | +/++/+++ | Relatively few labeled cells in layers 1 and 4; ++ in layer 2/3, 6a; +++ in layer 5. |
|  | Anterior cingulate area (ACA) | ++/+++ | Many labeled cells present. Most moderately labeled, but stronger in layer 5. |
|  | Prelimbic area (PL) | ++/+++ | Many labeled cells present. Most moderately labeled, but stronger in layer 5. |
|  | Orbital Area (ORB) | ++/+++ | Numerous labeled cells throughout cellular layers |
|  | Agranular insular area (AI) | ++/+++ | Numerous labeled cells throughout cellular layers. Layer 2/3 more strongly labeled than other layers |
|  | Retrosplenial area (RSP) | ++ | Abundant labeled cells, but not as strongly labeled as other neocortical regions. |
|  | Posterior parietal association areas (PTLp) | +,++,+++ | Relatively few labeled cells in layers 1 and 4; ++ in layer 2/3, 6a; +++ in layer 5. |
|  | Ectorhinal area (ECT) | + | Many weakly labeled cells present. |
|  |  |  |  |
| Olfactory Areas (OLF) | Main olfactory bulb (MOB) | ++/+++ | Strong ELOVL4 labeling in cells around glomeruli and in mitral layer. Cells in granule cell layer show little labeling. |
|  | Accessory olfactory bulb (AOB) | +++ | Strongly labeled cells in the granular layer |
|  | Anterior olfactory nucleus (AON) | ++ | Prominent ELOVL4 labeling in layer 2, less labeling in layer 1. |
|  | Taenia tecta (TT) | ++ | Labeled cells present. |
|  | Piriform area (PIR) | ++ | Many moderately labeled cells in layer 2; labeled cells at lower density in layer 3. |
|  | Nucleus of lateral olfactory tract (NLOT) | +/++ | Weakly to moderately labeled cells present |
|  |  |  |  |
| Hippocampal formation (HF) |  |  |  |
| Hippocampus proper (HIP) |  |  |  |
| Cornu ammonis (CA) | CA1 | +/++ | Cells in CA1 show uniform, weak to moderate ELOVL4 labeling |
|  | CA2 | +/++ | Cells in CA2 show uniform, weak to moderate ELOVL4 labeling. Comparable to CA1. |
|  | CA3 | +++ | Cells in CA3 show strong ELOVL4 labeling. |
| Dentate gyrus (DG) |  |  |  |
|  | Dentate gyrus (DG) granule cell layer | -/+ | Little to no labeling in mature granule cells. Moderately labeled cells present along the inner margin of the granule cell layer. |
|  | Dentate gyrus (DG) molecular layer | -/+ | Very little ELOVL4 labeling. Only a few ELOVL4 positive cells |
|  | Dentate gyrus (DG) polymorphic layer | -/+++ | Many cells are ELOVL4 negative, but some cells in this area show strong ELOVL4 labeling. |
| Retrohippocampal region (RHP) | Entorhinal area (ENT) | ++ | Moderate ELOVL4 labeling present throughout this region. |
|  | Postsubiculum (POST) | ++/+++ | Many cells show moderate to strong ELOVL4 labeling. |
|  | Presubiculum (PRE) | ++/+++ | Many cells show moderate to strong ELOVL4 labeling. |
|  | Subiculum (SUB) | +++ | Many, but not all, cells show strong ELOVL4 labeling . |
|  |  |  |  |
| Cortical Subplate (CTXsp) |  |  |  |
|  | Isocortex, layer 6b (6b) | ++/+++ | Many cells show moderate ELOVL4 labeling, but cells adjacent to corpus callosum often display strong ELOVL4 labeling. |
|  | Claustrum (CLA) | +/++ | Numerous weakly to moderately ELOVL4+ cells present. |
|  | Endopiriform nucleus (EP) | ++ | Moderately ELOVL4+ cells present at relatively low density |
|  |  |  | *For amygdala nuclei derived from cortical subplate see “Amygdala complex” below.* |
|  |  |  |  |
| Basal Ganglia |  |  |  |
| Striatum (dorsal) | Caudoputamen (CP) | -/+ | Little ELOVL4 labeling present. Very few labeled cells. |
| Striatum (ventral) | Nucleus accumbens (ACB) | -/+ | Little ELOVL4 labeling present. Very few labeled cells. |
|  | Fundus of striatum (FS) | -/+ | Little ELOVL4 labeling present. Very few labeled cells. |
|  | Olfactory tubercle (OT) | ++ | Many moderately labeled cells in layer 2; labeled cells at lower density in layer 3. |
| Striatum (medial) | Lateral septal nucleus (LS) | +/++ | Many weakly to moderately labeled cells present. |
|  | Septofimbrial nucleus (SF) | -/+ | Only a few weakly labeled cells |
| Striatum (caudal) | *For amygdala nuclei derived from caudal striatum see “Amygdala complex” below.* |  |  |
| Pallidum (dorsal) | Globus Pallidus external (GPe) | -/+ | Little ELOVL4 labeling present. Very few labeled cells. |
|  | Globus Pallidus internal (GPi) | -/+ | Little ELOVL4 labeling present. Few labeled cells present, but at higher density than globus pallidus external. |
| Pallidum (ventral) | Substantia Innominata (SI) | -/+ | Only a few weakly labeled cells present |
|  | Magnocellular Nucleus (MA) | -/+ | Only a few weakly labeled cells present |
| Pallidum (medial; Medial Septal Complex) | Medial Septal Nucleus (MS) | ++/+++ | Many moderately to strongly labeled cells present. |
|  | Diagonal Band Nucleus (NDB) | +/++ | A few weakly and moderately labeled cells present. |
|  | Triangular Nucleus of Septum (TRS) | + | A few labeled cells present. |
| Pallidum (caudal) | Bed Nucleus of Stria Terminalis (BST) | + | A few labeled cells present. |
|  | Bed Nucleus of Anterior Commissure (BAC) | + | A few labeled cells present. |
|  |  |  |  |
| Amygdala complex |  |  |  |
| *Derived from Cortical plate* | Cortical amygdalar area (COA) | ++ | Numerous moderately labeled cells. |
| *Derived from Cortical subplate* | Basolateral amygdalar nucleus (BLA) | ++ | Many moderately labeled cells. |
|  | Basomedial amygdalar nucleus (BMA) | ++ | Many moderately labeled cells. |
|  | Posterior amygdalar nucleus (PA) | +/++ | Moderately labeled cells present. Not as strongly labeled as some other regions of amygdala. |
| *Derived from Striatum* | Anterior amygdalar area (AAA) | +/++ | Moderately labeled cells present. Not as strongly labeled as some other regions of amygdala. |
|  | Central amygdalar nucleus (CEA) | ++/+++ | Many moderately to strongly labeled cells |
|  | Medial amygdalar nucleus (MEA) | +/++ | Moderately labeled cells present. Not as strongly labeled as some other regions of amygdala. |
|  |  |  | *Additional labeled cells are present in the Amygdala, but identification of specific nuclei containing labeled cells is not conclusive.* |
|  |  |  |  |
| **CEREBELLUM [Parencephalon] (CB)** |  |  |  |
| Cerebellar cortex (CBX) | Vermis: (Lingula; Central Lobules I, II and III; Culmen, lobules 4 and 5; Declive, lobule 6; Folium Tuber vermis, Lobule VII; Pyramus, lobule VIII; Uvula, lobule IX; Nodulus, lobule X)  Hemisphere: (Simple lobule, Ansiform lobule, Paramedian lobule, Copula pyramus, Paraflocculus, Flocculus) | ++/+++ | All lobules of cerebellar cortex show a similar labeling pattern: Sparsely distributed cells in the molecular layer show moderate to strong ELOVL4 labeling. Purkinje cells show moderate labeling for ELOVL4.Cells in the granule cell layer show very strong labeling for ELOVL4. No differences noted between lobules in the vermis and lobules in the hemispheres. |
| Cerebellar nuclei (CBN) | Fastigial nucleus (FN), Interposed nucleus (IP), Dentate nucleus (DN) | +++/++++ | All deep cerebellar nuclei showed a similar labeling pattern: Many cells distributed throughout the nucleus shows strong to intense ELOVL4 labeling, but many cells in the nucleus show no ELOVL4 labeling. |
|  |  |  |  |
| **DIENCEPHALON** |  |  |  |
| Thalamus (TH) |  |  |  |
| *Sensory-motor cortex-related nuclei* | Ventral-Anterior-Lateral complex (VAL) | +/++ | Weakly to moderately labeled cells present, not as dense as other thalamic regions. |
|  | Ventral medial nucleus (VM) | +/++ | Moderately ELOVL4+ cells present, not as dense as other thalamic regions. |
|  | Ventral posterolateral nucleus (VPL) | +/++ | Some cells show weak to moderate labeling. |
|  | Ventral posteromedial nucleus (VPM) | +/++ | Some cells show weak to moderate labeling. |
|  | Subparafascicular nucleus (SPF) | -/+ | Some cells show weak labeling. |
|  | Peripeduncular nucleus (PP) | -/+ | Few labeled cells present. |
|  | Medial geniculate nucleus (MG) | ++ | Numerous moderately ELOVL4+ cells |
|  | Lateral geniculate Nucleus (LGN) | ++/+++ | Numerous moderately to strongly ELOVL4+ cells present. |
|  |  |  |  |
| *Polymodal association cortex-related* | Lateral posterior nucleus (LP) | +/++ | Some cells show weak to moderate labeling. |
|  | Posterior complex of Thalamus (PO) | +/++ | Some weakly to moderately labeled cells present, but less intense than many other thalamic nuclei |
|  | Anteroventral nucleus (AV) | +++ | Many strongly labeled cells present. |
|  | Anteromedial nucleus (AM) | +++ | Many strongly labeled cells present. |
|  | Anterodorsal nucleus (AD) | +++/++++ | Many strongly labeled cells present. AD often is among most intensely labeled nuclei in entire brain. |
|  | Lateral dorsal nucleus (LD) | ++/+++ | Many moderately to strongly labeled cells present. |
|  | Mediodorsal nucleus (MD) | ++ | Many moderately labeled cells present. |
|  | Central medial nucleus (CM) | +++ | Many strongly labeled cells present. |
|  | Parafascicular nucleus (PF) | ++ | Many moderately labeled cells present. |
|  |  |  |  |
| *Reticular nucleus* | Reticular nucleus (RT) | ++ | Many moderately labeled cells present. |
|  |  |  |  |
| *Geniculate group of ventral thalamus* | Intergeniculate leaflet, lateral genicular complex (IGL) | ++/+++ | Many moderately to strongly labeled cells. |
|  | Ventral part of LGN, lateral zone (LGvl) | ++/+++ | Many moderately to strongly labeled cells. |
|  | Subgeniculate nucleus (SubG) | ++ | Many moderately labeled cells. |
|  |  |  | *Additional labeled cells are present in the Thalamus, but identification of specific nuclei containing labeled cells is not conclusive* |
|  |  |  |  |
| Hypothalamus (HY) |  |  |  |
| *Periventricular zone-Neuroendocrine motor zone* |  |  | *Labeled cells are present in this zone of the hypothalamus, but identification of specific nuclei containing labeled cells remains inconclusive.* |
| *Periventricular region* |  |  | *Labeled cells are present in this region of the hypothalamus, but identification of specific nuclei containing labeled cells remains inconclusive.* |
| *Hypothalamic medial zone - behavioral control column* | Anterior nucleus (AHN) | ++ | Many moderately to strongly labeled cells. |
|  | Mammilary body (MBO) | +/++ | Weak to moderately labeled cells present in lateral mammilary nucleus. Little or no labeling evident in Supramammilary nucleus (SUM). |
| *Hypothalamic lateral zone (LZ)* | Lateral hypothalamic area (LHA) | ++ | Many moderately labeled cells. |
|  | Lateral preoptic area (LPO) | ++ | Many moderately labeled cells. Labeling varies across nucleus. |
|  | Subthalamic nucleus (STN) | ++/+++ | Moderately to strongly labeled cells. |
|  | Zona incerta (ZI) | ++/+++ | Moderately to strongly labeled cells. |
|  |  |  |  |
| **MESENCEPHALON (Midbrain)** |  |  |  |
| *Sensory related* | Superior colliculus, sensory portion (SCs) | ++ | Many moderately labeled cells present. |
|  | Inferior colliculus (IC) | ++ | Many moderately labeled cells present. |
|  |  |  | *Additional labeled cells present in areas of the midbrain likely associated with sensory function, but identification of specific nuclei containing labeled cells is not conclusive.* |
|  |  |  |  |
| *Motor related* | Substantia nigra, pars reticulata (SNr) | -/++ | Little ELOVL4 labeling, but a few moderately labeled cells present. |
|  | Ventral tegmental area (VTA) | -/+ | Little to no ELOVL4 labeling |
|  | Midbrain reticular nucleus (MRN) | ++/+++ | Many moderately to strongly labeled cells. |
|  | Superior Colliculus, motor portion (SCm) | ++ | Many moderately labeled cells present. |
|  | Periaqueductal grey (PAG) | +/++/+++ | ELOVL4 distribution is not uniform across PAG. Numerous weakly to strongly labeled cells distributed in PAG. |
|  | Pretectal region (PRT) | +/++ | Weakly to moderately labeled cells present. |
|  | Red nucleus (RN) | -/++ | Most cells ELOVL4 negative, but a few moderately to strongly labeled cells present. |
|  |  |  | *Additional labeled cells present in areas of the midbrain likely associated with motor function, but identification of specific nuclei containing labeled cells is not conclusive.* |
|  |  |  |  |
| *Behavioral state related* | Substantia nigra, pars compacta (SNc) | -/++ | Little ELOVL4 labeling, but a few moderately labeled cells present. |
|  |  |  | *Additional labeled cells present in areas of the midbrain likely associated with behavioral state, but identification of specific nuclei containing labeled cells is not conclusive.* |
|  |  |  |  |
| **RHOMBENCEPHALON** |  |  |  |
| PONS (METENCEPHALON) |  |  |  |
| *Sensory related* | Principal sensory nucleus of the trigeminal (PSV) | ++/+++ | Appreciable number of moderately and strongly ELOVL4+ cells. Some strongly labeled cells are quite large. |
|  | Parabrachial nucleus (PB) | +/++ | Weakly to moderately labeled cells present. |
|  | Superior olivary complex (SOC) | -/++/+++ | Most cells show little or no labeling, but a few cells show moderate to strong labeling. |
|  |  |  | *Additional labeled cells present in areas of the Pons likely associated with sensory function, but identification of specific nuclei containing labeled cells is not conclusive.* |
|  |  |  |  |
| *Motor related* | Pontine central grey (PCG) | +/++ | Some weakly to moderately labeled cells present. |
|  | Pontine grey (PG) | +++/++++ | Many strongly to intensely labeled cells. |
|  | Pontine reticular nucleus, caudal (PRNc) | ++/+++ | Many moderately to strongly to labeled cells. Cluster of very strongly labeled cells in anterior portion of nucleus. |
|  | Tegmental reticular nucleus (TRN) | +++/++++ | Many strongly to intensely labeled cells. |
|  | Motor nucleus of the trigeminal (V) | +++ | Numerous strongly labeled cells present. |
|  |  |  | *Additional labeled cells present in areas of the Pons likely associated with motor function, but identification of specific nuclei containing labeled cells is not conclusive.* |
|  |  |  |  |
| *Behavioral state related* | Pontine reticular nucleus (PRNr) | +/++ | Many weakly to moderately labeled cells present. |
|  |  |  | *Additional labeled cells present in areas of the Pons likely associated with behavioral state, but identification of specific nuclei containing labeled cells is not conclusive.* |
|  |  |  |  |
| MYENCEPHALON (Medulla) |  |  |  |
| *Sensory related* | Cochlear nucleus, dorsal (DCO) | ++/+++ | Many moderately to strongly labeled cells present. |
|  | Cochlear nucleus, ventral (VCO) | ++ | Many moderately labeled cells. Less intense labeling than DCO. |
|  | Nucleus of the solitary tract (NTS) | +/++ | Many weakly to moderately labeled cells. |
|  | Spinal nucleus of the trigeminal (SPV) | +/++/+++ | Many labeled cells present. Labeling intensity varies across nucleus. |
|  |  |  | *Additional labeled cells present in areas of the Medulla likely associated with sensory function, but identification of specific nuclei containing labeled cells is not conclusive.* |
|  |  |  |  |
| *Motor related* | Gigantocellular reticular nucleus (GRN) | ++ | Numerous moderately ELOVL4+ cells |
|  | Intermediate reticular nucleus (IRN) | ++ | Numerous moderately ELOVL4+ cells |
|  | Lateral reticular nucleus (LRN) | +++ | Many strongly labeled cells present. |
|  | Magnocellular reticular nucleus (MARN) | ++ | Many moderately labeled cells present. |
|  | Medullary reticular nucleus (MDRN) | ++ | Many moderately labeled cells present. |
|  | Parvicellular reticular nucleus (PARN) | ++ | Many moderately labeled cells present. |
|  | Lateral vestibular nucleus (LAV) |  | *Labeled cells may be present in lateral vestibular nucleus, but identification is not conclusive.* |
|  | Medial vestibular nucleus (MV) | ++/+++ | Moderately to strongly labeled cells present. |
|  | Spinal vestibular nucleus (SPIV) | ++/+++ | Moderately to strongly labeled cells present. |
|  | Superior vestibular nucleus (SUV) | +/++ | Some weak to moderately labeled cells present. Less prominent labeling than medial and spinal vestibular nuclei. |
|  |  |  | *Additional labeled cells present in areas of the Medulla likely associated with motor function, but identification of specific nuclei containing labeled cells is not conclusive.* |
|  |  |  |  |
| *Behavioral state related* |  |  | *Labeled cells present in areas of the Medulla likely associated with behavioral state, but identification of specific nuclei containing labeled cells is not conclusive.* |
